# Dependence receptor UNC5A restricts luminal to basal breast cancer plasticity and metastasis

**DOI:** 10.1186/s13058-018-0963-5

**Published:** 2018-05-02

**Authors:** Maria B. Padua, Poornima Bhat-Nakshatri, Manjushree Anjanappa, Mayuri S. Prasad, Yangyang Hao, Xi Rao, Sheng Liu, Jun Wan, Yunlong Liu, Kyle McElyea, Max Jacobsen, George Sandusky, Sandra Althouse, Susan Perkins, Harikrishna Nakshatri

**Affiliations:** 1 Department of Surgery, Indiana University School of Medicine, Indianapolis, IN 46202 USA; 2 Department of Medical and Molecular Genetics, Indiana University School of Medicine, Indianapolis, IN 46202 USA; 3 Center for Computational Biology and Bioinformatics, Indiana University School of Medicine, Indianapolis, IN 46202 USA; 4 Department of Pathology and Laboratory Medicine, Indiana University School of Medicine, Indianapolis, IN 46202 USA; 5 Department of Biostatistics, Indiana University School of Medicine, Indianapolis, IN 46202 USA; 6 Department of Biochemistry and Molecular Biology, Indiana University School of Medicine, Indianapolis, IN 46202 USA; 7 VA Roudebush Medical Center, C218C, 980 West Walnut St, Indianapolis, IN 46202 USA; 8 Present Address: Department of Pediatrics and Herman B. Wells Center for Pediatrics Research, Indiana University School of Medicine, Indianapolis, IN USA

**Keywords:** Breast cancer, UNC5A, Netrin-1, Estrogen receptor, Estradiol and metastasis

## Abstract

**Background:**

The majority of estrogen receptor-positive (ERα^+^) breast cancers respond to endocrine therapies. However, resistance to endocrine therapies is common in 30% of cases, which may be due to altered ERα signaling and/or enhanced plasticity of cancer cells leading to breast cancer subtype conversion. The mechanisms leading to enhanced plasticity of ERα-positive cancer cells are unknown.

**Methods:**

We used short hairpin (sh)RNA and/or the CRISPR/Cas9 system to knockdown the expression of the dependence receptor *UNC5A* in ERα^+^ MCF7 and T-47D cell lines. RNA-seq, quantitative reverse transcription polymerase chain reaction, chromatin immunoprecipitation, and Western blotting were used to measure the effect of *UNC5A* knockdown on basal and estradiol (E2)-regulated gene expression. Mammosphere assay, flow cytometry, and immunofluorescence were used to determine the role of UNC5A in restricting plasticity. Xenograft models were used to measure the effect of *UNC5A* knockdown on tumor growth and metastasis. Tissue microarray and immunohistochemistry were utilized to determine the prognostic value of UNC5A in breast cancer. Log-rank test, one-way, and two-way analysis of variance (ANOVA) were used for statistical analyses.

**Results:**

Knockdown of the E2-inducible *UNC5A* resulted in altered basal gene expression affecting plasma membrane integrity and ERα signaling, as evident from ligand-independent activity of ERα, altered turnover of phosphorylated ERα, unique E2-dependent expression of genes effecting histone demethylase activity, enhanced upregulation of E2-inducible genes such as BCL2, and E2-independent tumorigenesis accompanied by multiorgan metastases. *UNC5A* depletion led to the appearance of a luminal/basal hybrid phenotype supported by elevated expression of basal/stem cell-enriched ∆Np63*,* CD44*,* CD49f, epidermal growth factor receptor (EGFR), and the lymphatic vessel permeability factor *NTN4*, but lower expression of luminal/alveolar differentiation-associated *ELF5* while maintaining functional ERα. In addition, *UNC5A*-depleted cells acquired bipotent luminal progenitor characteristics based on KRT14^+^/KRT19^+^ and CD49f^+^/EpCAM^+^ phenotype. Consistent with in vitro results, UNC5A expression negatively correlated with EGFR expression in breast tumors, and lower expression of UNC5A, particularly in ERα^+^/PR^+^/HER2^−^ tumors, was associated with poor outcome.

**Conclusion:**

These studies reveal an unexpected role of the axon guidance receptor UNC5A in fine-tuning ERα and EGFR signaling and the luminal progenitor status of hormone-sensitive breast cancers. Furthermore, *UNC5A* knockdown cells provide an ideal model system to investigate metastasis of ERα^+^ breast cancers.

**Electronic supplementary material:**

The online version of this article (10.1186/s13058-018-0963-5) contains supplementary material, which is available to authorized users.

## Background

The luminal subtypes that express the estrogen receptor (ER)α represent approximately 70% of breast cancers, and the majority of these tumors respond to endocrine therapy [1]. However, resistance to endocrine therapy resulting in relapse is seen in approximately 30% of patients [1]. ERα^+^ breast cancers are heterogeneous with at least two subtypes, luminal A and luminal B [2]. Luminal A tumors are estradiol (E2)-dependent and responsive to antiestrogens, whereas luminal B tumors display either intrinsic or acquired resistance to antiestrogens with an outcome almost similar to triple negative breast cancers (TNBCs) [3]. A subgroup of luminal A tumors, particularly those that have metastasized despite expressing luminal A biomarkers (ERα and progesterone receptor (PR)), do not respond to antiestrogen therapies and approximately 55% of these metastases have converted to a different subtype through an unknown mechanism [4].

Multiple mechanisms of antiestrogen resistance have been documented [5]. Most of the prior work focused on mechanisms that confer E2-independent activity to ERα, including kinases that phosphorylate ERα, co-activator molecules that enhance ERα activity, pioneer factors that govern chromatin binding of ERα, and growth factor receptor–ERα crosstalk [6–8]. However, to our knowledge, there have been limited attempts to decipher negative regulatory loops that may restrict ERα signaling subsequent to ligand-activated induction and deregulation of these negative regulatory loops leading to prolonged/sustained activation of ERα.

To identify luminal cell-expressed genes that may play a role in restricting E2-dependent proliferation, we scanned gene expression array datasets for E2-inducible genes with ERα binding sites and that have a growth inhibitory activity [9]. From this search, we focused on the dependence receptor (DR) pathways for their potential role in a negative feedback loop. Under physiological conditions, unliganded DRs elicit cell death and/or growth inhibition but elicit cell survival and proliferation when coupled with their ligands such as Netrin-1 (NTN1) [10]. DRs are direct transcriptional targets of p53 and integral to p53-dependent apoptotic pathways, particularly in the absence of ligands [11]. NTN1 belongs to the evolutionary conserved netrin family secreted proteins and is well characterized for its role in the nervous system [12]. Both netrins and DRs also play crucial roles in other systems, including development of the mammary gland, inner ear, lungs, and pancreas [12, 13]. Loss of heterozygosity and homozygous deletion of DRs and upregulation of netrins are observed in a variety of cancers including breast cancer [11, 13]. These aberrations in DR–netrin pathways are believed to confer resistance to p53-dependent apoptosis and enhance proliferation of cancer cells.

In the present study, we show that *UNC5A* is an E2-inducible gene. Knockdown of *UNC5A* in ERα^+^/PR^+^ cells resulted in defective turnover of phosphorylated ERα, enhanced E2 signaling, cell proliferation, and tumorigenesis independent of E2 supplementation accompanied with multiorgan metastases in xenograft models. Furthermore, *UNC5A* knockdown cells acquired a hybrid basal/luminal phenotype including elevated expression of epidermal growth factor receptor (EGFR). Thus, UNC5A could serve as a negative feedback molecule in ERα signaling, the deregulation of which could lead to breast cancer progression through enhanced plasticity.

## Methods

### Immunohistochemistry of tissue microarray (TMA)

Tissue samples were collected with Indiana University Institutional Review Board approval, informed patient consent, and HIPAA compliance. UNC5A and EGFR immunostaining was performed at the CLIA certified Indiana University Health Pathology Laboratory and scoring has been described previously [14]. *H* scores were calculated using stain intensity (0 to 3) multiplied by percent positive pixels (for UNC5A) or a formula based on stain intensity and number of weak, moderate, or strong positive pixels (for EGFR). For subjects with multiple tumor samples, only those with the highest *H* score were considered. Statistical analysis was performed on samples from 221 breast cancer patients, but only 196 patient samples (89%) had UNC5A values available. The log-rank test was used to compare patient and tumor variables between those with UNC5A *H* scores versus those without. The correlations between UNC5A and EGFR were determined by Spearman’s correlation coefficient. For modeling the outcomes of overall survival and disease-free survival, the multivariate covariates used in the multivariate models from the individual reports for EGFR and UNC5A were included. Additionally, the *H* score information for EGFR and UNC5A were handled in three ways. First, the EGFR and UNC5A were dichotomized using the same optimal cut-points as used in their individual reports. Secondly, the EGFR and UNC5A were dichotomized using their individual medians and cut-points. Finally, the continuous values were used in the models. Since EGFR was not linear, the natural log of EGFR was used in the models. For the models with continuous values, hazard ratios were calculated at the 25th, 50th, and 75th percentile of EGFR. Subgroup analyses were performed where the number of patients available was sufficient.

### Cell lines

MCF7 and T-47D cells were obtained from American Tissue Culture Collection and cultured in minimum essential media (MEM) media as described previously [15]. TMCF7 cells correspond to cell lines derived from tumors developed in the mammary fat pad of nude mice implanted with MCF7 cells [16]. Cell lines were authenticated using Short Tandem Repeat Profiling Systems for cell line identification by a commercial vendor (DNAcenter.com) in August 2012 and cell lines recreated from xenograft tumors were authenticated by Genetica (Burlington, NC, USA).

### Short hairpin (sh)RNA and CRISPR constructs

The human *shRNA* lentiviral transduction particles for *sh5-UNC5A* and pLKO.1-puro vector control plasmids (*sh-Control*) were purchased from Sigma (cat. nos. SHCLNV-NM_133369 and SHC 001, respectively). The lentivector for *sh2-UNC5A* was obtained from Applied Biological Materials (cat. no. i026703g). CRISPR plasmids to target *UNC5A* were obtained from Sigma-Aldrich (HS0000509914).

### Western blotting

Treatments consisted vehicle, heregulin-β1 (HRG-β1, R&D systems), E2, 4-hydroxy-tamoxifen (OHT), or ICI-182,780 (Sigma-Aldrich). The immunoblotting has been previously described [17] and details of antibodies are provided in Additional file 1. Although the majority of immunoblots were reprobed with antibodies against ACTB (β-actin) as a loading control, only representative data per batch of cell lysates are shown.

### RNA-seq and quantitative reverse transcription polymerase chain reaction (qRT-PCR)

cDNA was synthesized from 1 to 2 μg of total RNA using the cDNA Synthesis Kit (Bio-Rad). qRT-PCR in duplicates from at least two biological replicates was performed with either Sybr-Green or TaqMan Universal PCR master mix and transcripts were analyzed in StepOnePlus and TaqMan 7900HT instruments (Applied Biosystems) with *β-ACTIN* as the normalization control. Fold-change was calculated by the ∆∆Ct method, whereas statistical analysis was performed on ΔCt values. Primers (Integrated DNA Technologies) and TaqMan probe details are shown in Additional file 1. RNA-seq of *sh-Control* and *sh5-UNC5A* cells treated for 3 h with vehicle or E2 was performed in triplicate as previously described [18], and raw sequencing data have been submitted to the gene expression omnibus (GEO; accession number GSE89700).

We used STAR RNA-Seq aligner to map all sequence libraries to the human genome (UCSC hg19) [19] followed by the assignment of uniquely mapped reads to individual genes based on annotation of hg19 refGene by featureCounts [20]. After trimmed mean of M values (TMM) normalization, gene expression profiling was summarized on the base-2 logarithmic scale. Genes with an average expression level lower than 1 for all phenotypes in MCF7 and T-47D cells, respectively, were excluded for further analysis. Differential expression (DE) analysis was performed using edgeR [21, 22] for special group comparisons in the study. All *p* values were corrected by multiple testing false discovery rate (FDR) adjustments. Genes with FDR < 0.05 and absolute value of fold change (FC) larger than 2 were determined as differentially expressed genes (DEGs).

Gene function enrichment analysis was performed using DAVID (http://david.abcc.ncifcrf.gov/home.jsp v6.8) [23, 24]. Significantly overrepresented gene ontology (GO) terms were selected if their *q* values (*p* values after FDR multiple test correction) were less than 0.05.

### Promoter luciferase assay

Cells transfected with luciferase constructs were allowed to grow overnight in charcoal-dextran treated fetal calf serum (CCS) containing media followed by a 12-h E2 treatment. The Dual-Luciferase® Reporter assay (Promega) was performed according to the manufacturer’s protocol.

### Chromatin immunoprecipitation (ChIP) assay

ChIP assays for ERα binding on *UNC5A* and *BCL2* were performed under vehicle or E2 treatment for 45 min and 2 h as described previously [9].

### Cell proliferation and mammosphere assays

After 24-h plating in regular media, the media was changed to CCS-containing media for 3 days and cells were treated with the indicated drug combinations. Cell proliferation was determined using the bromodeoxyuridine-incorporation enzyme-linked immunosorbent assay (ELISA) kit from Calbiochem after 5–6 days of plating with one media/drug change. Mammosphere assays with 5000 cells were performed as described previously [25].

### Immunofluorescence

Cells grown on 35-mm glass-bottom culture dishes were fixed with 4% (w/v) paraformaldehyde for 10 min and permeabilized in phosphate-buffered saline (PBS) containing 0.15% (v/v) Triton X-100, 5% (v/v) donor goat serum (Gibco), and 1% (w/v) bovine serum albumin (Sigma-Aldrich) for 1 h. Cells were incubated with primary antibodies (Additional file 1) diluted in Dako antibody diluent (Dako; Agilent Technologies) for 90 min followed by 1-h incubation with the Alexa Fluor® 488 and 555 conjugated secondary antibodies (ThermoFisher Scientific). Nuclei was counterstained with Hoechst® 33,342.

### Flow cytometry

Cells were stained with the indicated antibodies (Additional file 1) and analyzed in a LSR4 custom-made flow cytometer (BD Biosciences) as described previously [26].

### Xenograft studies

The Indiana University Animal Care and Use Committee approved the use of animals in this study and all procedures were performed as per NIH guidelines. *sh-Control*, *sh2-UNC5A*, and *sh5-UNC5A* TMCF7 cells (2 × 10^6^ in 100 μl serum-free HBSS) were implanted into the mammary fat pad of 7-week-old female nude mice with or without a 60-day slow-release E2 pellet. Tumor growth was measured weekly and tumor volume was calculated as described previously [16]. After 12 weeks, the lungs and primary tumors were collected and processed for hematoxylin and eosin (H&E) and PECAM1 (CD31) staining. The whole slide digital imaging system of Aperio (ScanScope CS) was used for imaging of PECAM1-stained tumors. For the metastatic model, mice were inoculated with 2 × 10^5^ TMCF7 cells into the left cardiac ventricle. Ovaries, spleen, and adrenal glands were collected within 17 weeks and processed as described above.

### Statistical analysis

Statistical analyses were performed in GraphPad Prism® (6.02 version) or Statistical Analysis System (SAS; version 9.4) software with *p* < 0.05 considered as significant.

## Results

### UNC5A is a luminal cell-enriched gene and is E2 inducible

**Fig. 1:**
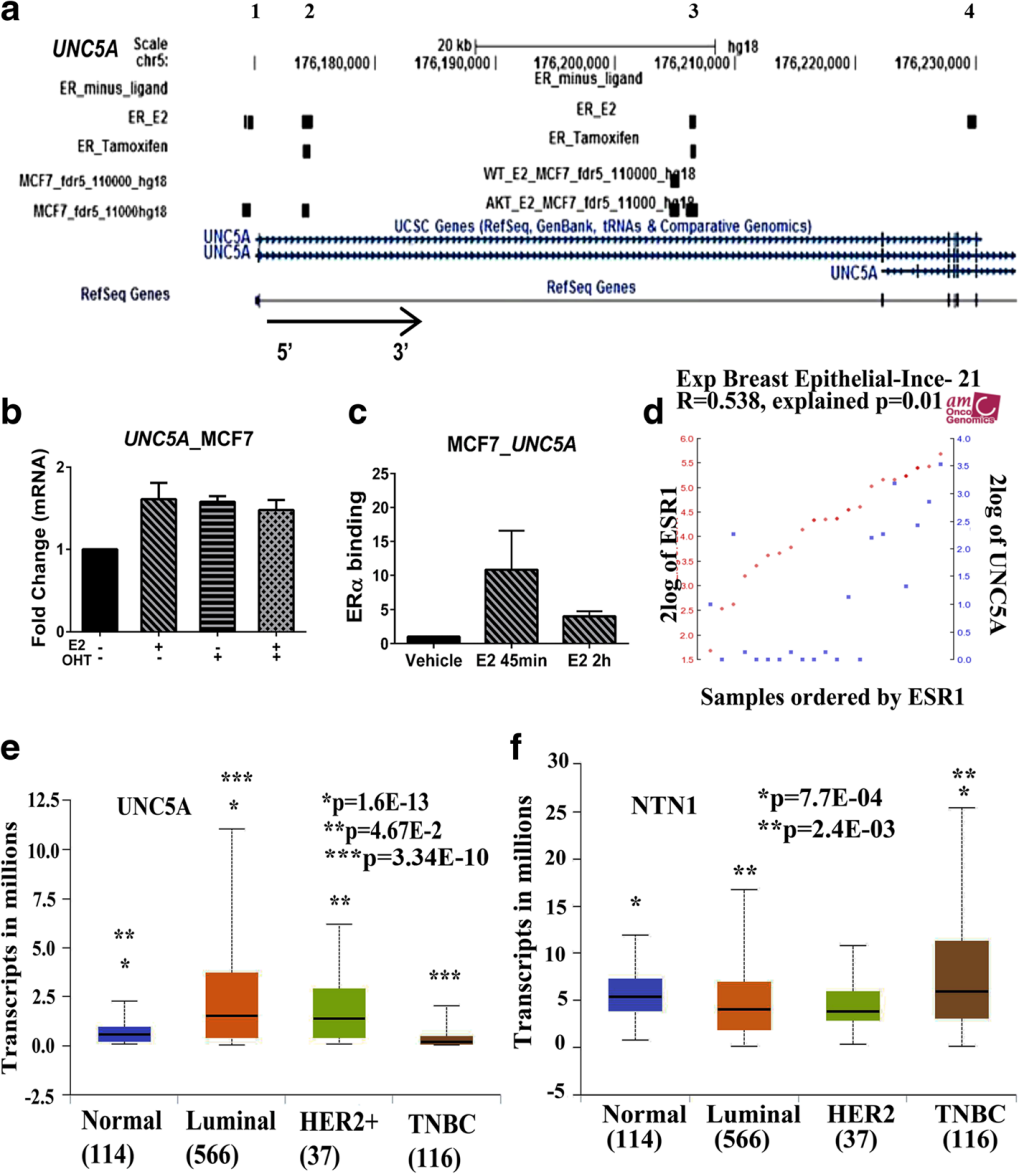
*UNC5A* is an estradiol (E2)-inducible gene. **a** Estrogen receptor (ER)α binding sites on *UNC5A* genomic region. Chromatin immunoprecipitation (ChIP)-seq datasets in MCF7 cells from Welboren et al. [27] (ER_minus ligand, ER-E2, ER-tamoxifen) and ChIP-on-chip datasets in MCF7 and MCF7 cells overexpressing constitutively active AKT from Bhat-Nakshatri et al. [9] were used to identify ERα binding sites on *UNC5A* genomic regions. Four ERα binding sites on chromosome 5 (hg18/human) are indicated on the top with genomic coordinates 176,169,194–176,169,471, 176,173,985–176,174,876, 176,206,209–176,206,749 and 176,229,374–176,230,036, respectively. **b** The effect of E2 (10^−10^ M), tamoxifen (OHT; 1 μM), or both on *UNC5A* expression in MCF7 cells. Cells were treated for 3 h and *UNC5A* levels were measured by qRT-PCR. Bar graphs represent mean ± SEM of fold change relative to vehicle control (*n* = 3). **c** ChIP assay confirms binding of ERα to *UNC5A* regulatory regions (second ERα binding site from the left). MCF7 cells were treated with vehicle or E2 (10^−8^ M) for 45 min and 2 h. ERα DNA binding levels are presented as mean ± SEM of non-normalized values relative to the vehicle *sh-Control* (*n* = 2). **d** mRNA levels of *ESR1* and *UNC5A* show positive correlation in breast cancer cell lines. R2 Genomics Analysis and Visualization Platform (http://r2.amc.nl) tool was used to obtain these results. **e** *UNC5A* mRNA levels in different subtypes of breast cancers in the TCGA dataset. Data were obtained using the public database UALCAN [28]. **f** *NTN1* mRNA levels in different subtypes of breast cancers in the TCGA dataset. TNBC, triple negative breast cancer

To determine E2-inducible signaling molecules that may dampen the E2 response or gene-specific E2 regulation and that are expressed at higher levels in luminal breast cancers compared with TNBCs, we first searched our previous microarray data of E2-regulated genes in MCF7 cells for known growth suppressive roles and then determined whether E2 directly regulated their expression by integrating E2-inducible gene expression with ERα ChIP-on-chip and ChIP-seq datasets. *UNC5A* suited these criteria as its expression was E2 inducible and ERα binding sites for this gene was detectable in ChIP-on-chip and ChIP-seq datasets [9, 27] (Fig. 1a). Furthermore, in 11 out of 12 studies in publicly available Nuclear Receptor Signaling Atlas web resources showed 2- to 35-fold E2-inducible expression of *UNC5A* in MCF7 cells, uterus, and vagina (Additional file 2). We further confirmed E2-inducible expression of *UNC5A* in MCF7 cells by qRT-PCR (Fig. 1b) and Western blotting (see below), although induction at mRNA levels in our MCF7 cells was modest. Interestingly, the antiestrogen tamoxifen (OHT) failed to overcome the effect of E2 on UNC5A levels (Fig. 1b) suggesting unique effects of E2 on the expression of UNC5A. We used ChIP assay to verify ERα binding to one of the ERα binding sites (Fig. 1c). R2 Genomic and Visualization Platform (http://r2.amc.nl) analyses revealed a positive correlation between *UNC5A* and *ESR1* mRNA levels in breast cancer cell lines (Fig. 1d). Also, analyses of The Cancer Genome Atlas (TCGA) dataset for the relationship between UNC5A expression and breast cancer subtypes using the UALCAN program [28] revealed highest UNC5A expression in luminal breast cancers, which are usually ERα-positive, compared with TNBCs (Fig. 1e). In contrast, NTN1 expression was higher in TNBCs compared with normal breast or luminal breast cancers (Fig. 1f).

### Low UNC5A expression in primary breast cancers is associated with poor outcome

**Fig. 2:**
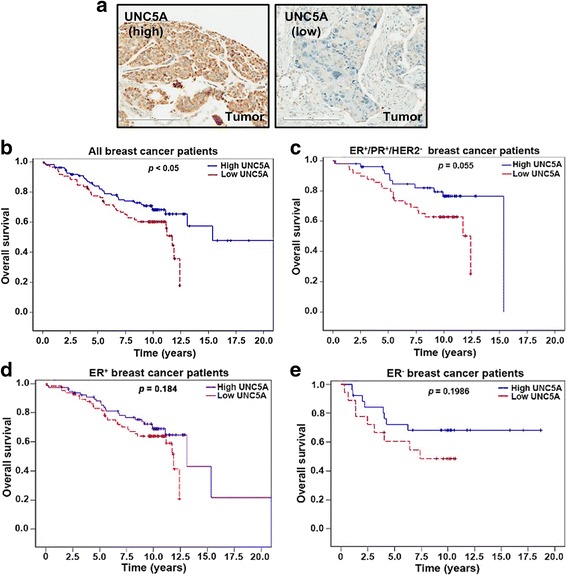
Low UNC5A in breast tumors correlates with poor overall survival of breast cancer patients. **a** Representative UNC5A staining pattern in breast tumors with high or low expression (scale bars = 200 μm). **b** Kaplan-Meier survival curves comparing overall survival of breast cancer patients with high (blue line) against low (red line) expression of UNC5A in tumors. Datasets were analyzed by log rank test from *n* = 196 patients. **c** Kaplan-Meier survival analysis of ER^+^/PR^+^/HER^−^ breast cancer patients according to the expression of UNC5A in tumors (*p* = 0.055) (*n* = 98). **d** Kaplan-Meier survival analysis of estrogen receptor (ER)α^+^ breast cancer patients according to the expression of UNC5A in tumors (*n* = 144). **e** Kaplan-Meier survival analysis of ERα^−^ breast cancer patients according to the expression of UNC5A in tumors (*n* = 44). PR, progesterone receptor

To obtain additional support for our hypothesis that a protein that attenuates ERα signaling has prognostic relevance, we performed immunohistochemical analyses of UNC5A in our previously described breast TMA in which 196 out of 221 tumors had measurable UNC5A expression [14] (Additional file 3). A representative staining pattern of UNC5A in breast tumor is shown in Fig. 2a. Tumor cells were moderate in staining in many of the cases with little to no background staining in the other tissues in the core (vascular endothelial cells, smooth muscle cells, fibroblasts, macrophages, and/or scattered lymphocytes infiltrating the tumor region). In both univariate and multivariate analyses, low UNC5A *H* score was associated with poor overall survival (Fig. 2b and Additional file 4). In subgroup analyses, in ER^+^/PR^+^/HER2^−^, lower UNC5A *H* score showed a trend of poor overall survival (*p* = 0.055) (Fig. 2c). UNC5A had no prognostic relevance when tumors were subgrouped broadly into ER^+^ or ER^−^ subgroups (Fig. 2d, e). Thus, UNC5A is a potential biomarker of outcome in a subgroup of breast cancer patients whose tumors express luminal A markers.

### *UNC5A* knockdown results in enhanced ERα signaling

**Fig. 3:**
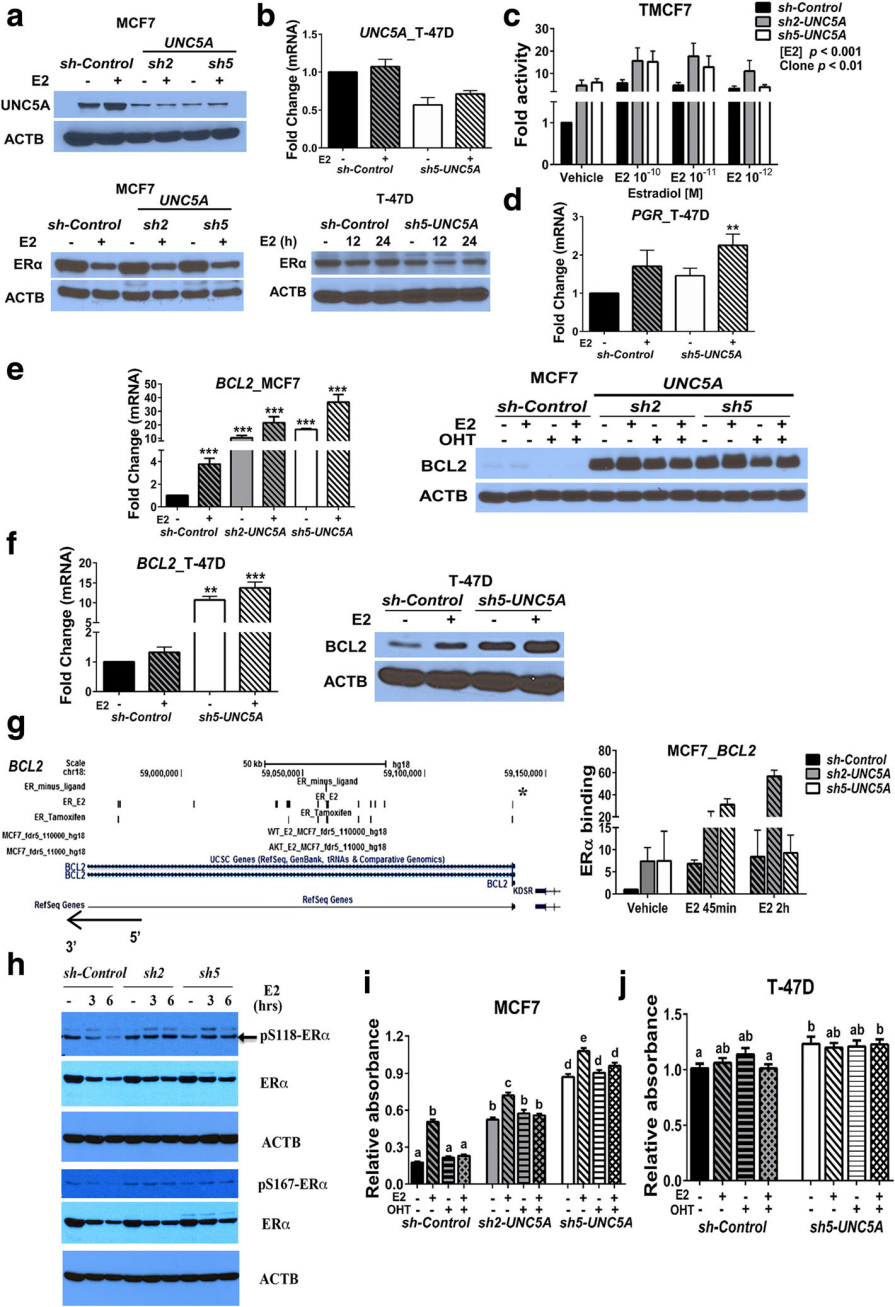
Estradiol (E2)-regulated gene expression and response in cells on *UNC5A* knockdown. **a** UNC5A expression in *sh-Control* and *sh-UNC5A* transfected MCF7 cells (top). *sh2-RNA* and *sh5-RNA* target independent sequences of *UNC5A*. E2-inducible expression of UNC5A protein is evident in *sh-Control* but not in *sh-UNC5A* transfected MCF7 cells. Estrogen receptor (ER)α expression in *sh-Control* and *sh-UNC5A* MCF7 cells treated with or without E2 for 24 h (bottom). **b** Generation of T-47D cells expressing *sh-UNC5A* (top). shRNA expressing cells have lower *UNC5A* transcripts (mean ± SEM; *n* = 3). As in MCF7 cells, *sh-UNC5A* had no effect on ERα protein levels in T-47D cells (bottom). **c** ERE-luciferase activity in *sh-Control* and *sh-UNC5A* TMCF7 cells. Cells were treated with vehicle or three different concentrations of E2 for 12 h (mean ± SEM; *n* = 4). Data were analyzed by two-way ANOVA where the main effects clone and [E2] were considered significant at *p* < 0.01 and *p* < 0.001, respectively. Note that ERE-luciferase activity was higher in *sh-UNC5A* clones in the absence of E2 treatment, although there was experimental variability. **d** *UNC5A* knockdown increases E2-inducible *PGR* expression in T-47D cells. Cells were treated with vehicle or E2 for 3 h (*n* = 4; **p < 0.01). **e** *UNC5A* knockdown leads to increased BCL2 expression. *BCL2* mRNA (left) was measured in vehicle and E2-treated (3 h) *sh-Control* and *sh-UNC5A* MCF7 cells (***p < 0.001). BCL2 protein levels were measured by Western blotting (right) in cells treated with vehicle, E2 (10^−10^ M), tamoxifen (OHT; 10^−6^ M), or an E2 and OHT combination for 24 h. **f** The effects of *UNC5A* knockdown on BCL2 expression in T-47D cells. Cells were treated with vehicle control and E2 for 3 h (mRNA) or 24 h (protein) (**p < 0.01 and ***p < 0.001). **g** *sh-UNC5A* enhances ERα binding to ERE-elements of *BCL2* in MCF7 cells. ERα binding sites on *BCL2* genomic regions identified using ChIP-seq and ChIP-on-chip data are shown in the left. ERα binding to ERE-elements (right most binding site indicated by a star in the ChIP-seq dataset, genomic coordinates, Chr18; 59,136,368–59,136,898) of BCL2 was verified by ChIP-qPCR assay (mean ± SEM of non-normalized values relative to vehicle *sh-Control; n* = 2). ERα binding in vehicle-treated *sh-Control* cells was set at 1 and the relative difference in other conditions is shown. **h** The effect of UNC5A knockdown on phosphorylated ERα. Cells were treated with E2 for 3 h or 6 h and the cell lysates were analyzed for ERα phosphorylated at S118 or S167 and total ERα. While phosphorylated ERα underwent activation-coupled degradation on E2 treatment in *sh-Control* cells, phosphorylated ERα was refractory to degradation in *sh-UNC5A* cells. **i** The effect of *UNC5A* knockdown on proliferation of *sh-Control* and *sh-UNC5A* MCF7 cells. Cells were treated for 5 days with vehicle control, E2, OHT, or E2 + OHT. Data are presented as mean of relative absorbance ± SEM (*n* = 2, each with six technical replicates) and were analyzed by ANOVA. Bars with the same character/letters are not significantly different according to Tukey’s test. For example, E2-induced proliferation rate of *sh-Control* cells is similar to the proliferation rate of vehicle-treated *sh2-UNC5A* cells. **j** The effect of *UNC5A* knockdown on proliferation of T-47D cells. Assays were performed as in **i** and the statistical results are presented as in **i**

To model low *UNC5A* levels in cells with intact ERα-dependent signaling, we created *shRNA-UNC5A* MCF7 and T-47D cells, which express ERα and PR at different levels (Fig. 3a, b). MCF7 cells are more responsive to E2 than T-47D cells and, therefore, most of the experiments were performed in MCF7 cells with a few validation experiments in T-47D cells. *UNC5A* knockdown did not have an effect on ERα levels and the receptor underwent activation-coupled degradation upon E2 treatment in both *sh-Control* and *sh-UNC5A* cells (Fig. 3a, b). Note that E2 increased UNC5A protein in *sh-Control* but not in *sh-UNC5A* MCF7 cells (Fig. 3a). We note that this is the only commercially available antibody (Abcam, ab81165) that recognized protein of expected size but showed variability in potency between batches. In transient transfection assay, estrogen response element (ERE)-driven luciferase-reporter gene showed elevated activity in vehicle-treated *sh-UNC5A* cells compared with *sh-Control* cells (Fig. 3c). In T-47D cells, which express higher levels of PR than MCF7 cells [29], *UNC5A* knockdown enhanced E2-inducible expression of *PGR* (Fig. 3d). In both MCF7 and T-47D cells, *UNC5A* knockdown substantially increased both basal (vehicle-treated) and, consequently, E2-inducible expression of BCL2 mRNA and protein (Fig. 3e, f). The enhanced BCL2 expression in *sh-UNC5A* cells correlated with increased E2-independent binding of ERα to the enhancer element of *BCL2* to which ERα and JMJD3 bind to create a poised chromatin [30] (Fig. 3g). To determine whether enhanced ERα activity in *UNC5A*-knockdown cells is due to altered phosphorylation of ERα, we used phospho-specific antibodies to determine the levels of ERα phosphorylated at S118 and S167. Phosphorylation of ERα at these residues is known to confer ligand-independent activity to the receptor [1]. Although we did not find any differences in basal phosphorylation status between *sh-Control* and *sh-UNC5A* cells, phosphorylated ERα underwent ligand-coupled degradation in *sh-Control* cells but not in *sh-UNC5A* cells (Fig. 3h). As a consequence, there was a modest difference in the rate of degradation of total ERα between clones. These results suggest the need for signaling events downstream of *UNC5A* in turnover of phosphorylated ERα.

To determine whether the above observations of altered phospho-ERα turnover upon UNC5A knockdown show any relationship with cell proliferation, we measured cell proliferation under vehicle control and E2 ± OHT-treated conditions. In MCF7 cells, *sh-UNC5A* increased proliferation under vehicle control, E2-, OHT-, and OHT plus E2-treated conditions compared with *sh-Control* to levels similar to E2-treated *sh-Control* cells (Fig. 3i, j). Although OHT reduced E2-inducible proliferation of *sh-UNC5A* cells, the overall proliferation rate of these cells under various treatments remained elevated compared with *sh-Control* cells. Collectively, these results suggest that UNC5A restricts the proliferation of ERα-positive cells.

To investigate whether enhanced the baseline proliferation of *sh-UNC5A* cells compared with *sh-Control* cells is ERα-dependent, we treated cells with ICI-182,780 (Fulvestrant) which degrades ERα. While ICI-182,780 reduced E2-induced proliferation of these cells, it had a minimal effect on baseline proliferation of all cell types (Additional file 5). These negative results can be interpreted in two ways: enhanced basal proliferation of *sh-UNC5A* cells compared with *sh-Control* cells is independent of ERα, or ERα in *sh-UNC5A* cells is less sensitive to ICI-182780-mediated degradation. Surprisingly, although ICI-182,780 caused degradation of total ERα in *sh-Control* and *sh-UNC5A* cells to a similar extent, ICI-182,780 increased the levels of ERα phosphorylated at S118 (Additional file 5). This unique effect of ICI-182,780 on phospho-ERα could explain the lack of its effects on the baseline proliferation rate of *sh-UNC5A* cells. Additional work is needed to clarify the role of ERα in the baseline proliferation rate of *sh-UNC5A* cells.

The dramatic effect of UNC5A on BCL2 expression was puzzling. To ensure that this increase in BCL2 expression is not due to aberrant integration of shRNAs into the genome, we used the CRISPR/Cas9 system to reduce *UNC5A* expression and selected single cell clones (Additional file 5). UNC5A protein levels were partially reduced in these single cell clones with an accompanying increase in BCL2 expression. Thus, even a modest decrease in UNC5A protein levels was sufficient to trigger BCL2 expression. We also observed stable BCL2 overexpression in both *UNC5A* shRNA and CRISPR clones cultured for a prolonged time despite these clones regaining UNC5A protein expression as measured using the available antibody. Thus, it appears that even transient knockdown of *UNC5A* leads to robust/permanent activation of *BCL2*, which is similar to previously reported stable activation of cancer germline genes upon transient knockdown of DNA methyltransferase 1 (DNMT1) [31].

### Changes in gene expression associated with *UNC5A* knockdown

**Fig. 4:**
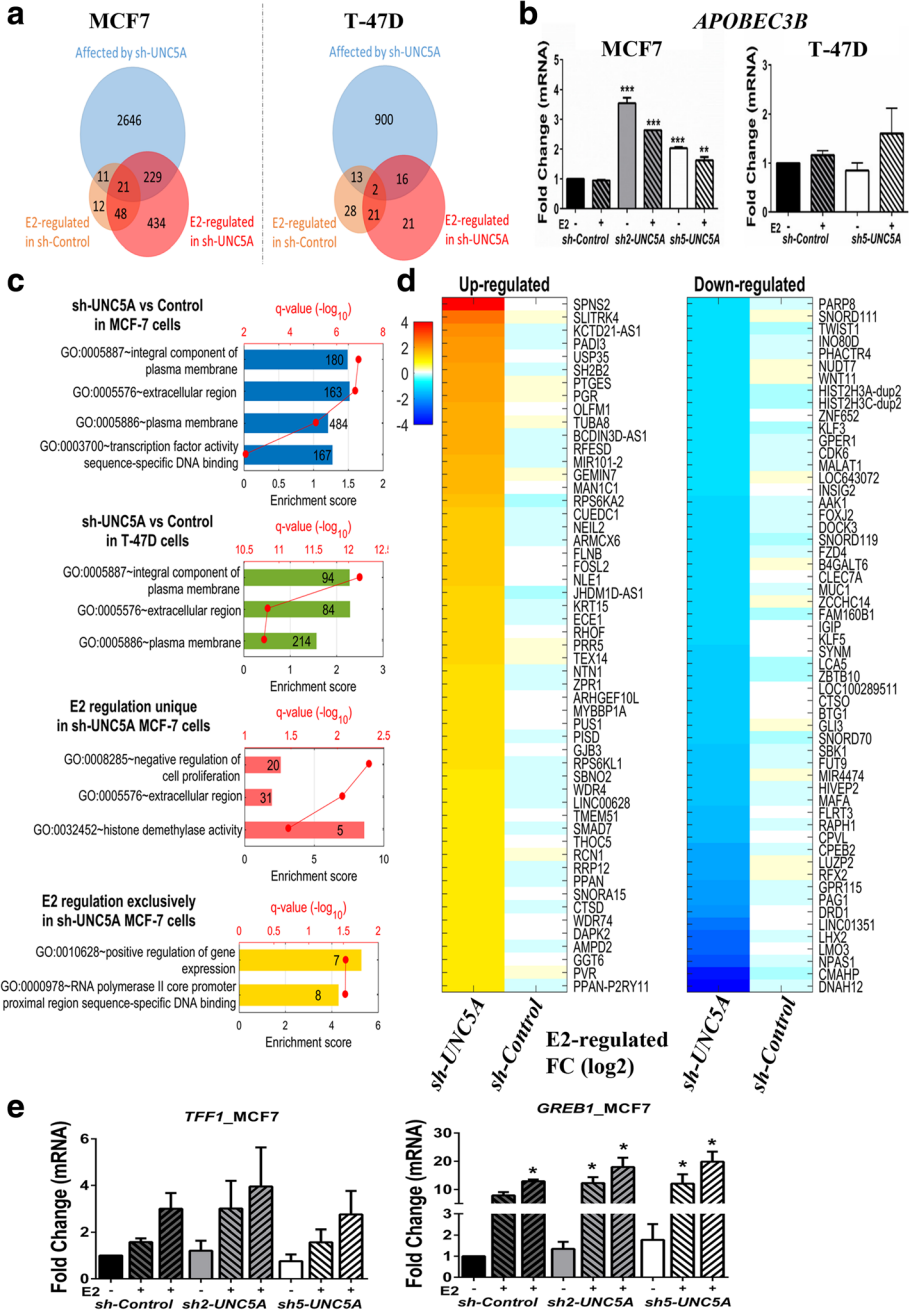
The effect of UNC5A knockdown on basal and estradiol (E2)-regulated gene expression in MCF7 and T-47D cells. **a** Venn diagram showing the number of differentially expressed genes in *UNC5A* knockdown MCF7 and T-47D cells compared with *sh-Control* cells with and without 3 h E2 treatment. **b** *UNC5A* knockdown MCF7 but not T-47D cells express higher levels of *APOBEC3B* (**p < 0.01 and ***p < 0.001). **c** Signaling pathways affected by UNC5A knockdown under vehicle and E2-treated conditions. The number of genes in each of the networks is shown and name of the genes in each network and fold changes in expression are presented in Additional files 7 and 6, respectively. UNC5A affected mainly basal gene expression in T-47D cells. The gene set ‘E2 regulation unique in *sh-UNC5A* MCF7 cells’ corresponds to those genes whose magnitude of E2-regulated expression differed from *sh-Control* cells, although these genes may be E2-regulated in both cell types. The gene set ‘E2 regulation exclusively in *sh-UNC5A* MCF7 cells’ corresponds to those genes whose expression was E2-regulated only in *sh-UNC5A* cells. **d** Heatmap of genes uniquely upregulated or downregulated by E2 in *sh-UNC5A* MCF7 cells. E2 did not affect their expression in *sh-Control* MCF-7 cells. **e** The effect of UNC5A knockdown on basal and E2-inducible expression of TFF1 and GREB1. TFF1 and GREB1 expression was measured by qRT-PCR in vehicle, 3-h, and 6-h E2-treated cells. GREB1 expression in 3-h and 6-h E2 treated *sh-UNC5A* cells was modestly but significantly higher compared with E2-treated *sh-Control* cells (indicated by *)

We performed RNA*-*seq of *sh-Control* and *sh5-UNC5A* MCF7 and T-47D cells treated with vehicle (basal) or E2 for 3 h and did pairwise comparisons to determine the effect of UNC5A on basal and E2-regulated gene expression (Fig. 4a). Genes were determined as DEGs for comparison if their FDR was < 0.05 and absolute value of fold change |FC| was > 2 (Fig. 4a and Additional file 6). Under basal growth conditions, *UNC5A* knockdown notably affected the expression of approximately 20% and 7% of genes in MCF7 and T-47D cells, respectively, potentially indicating its role in regulating the transcriptional machinery. For example, *APOBEC3B*, which is integral to ERα signaling [32], was one of the genes differentially expressed in *sh-UNC5A* cells compared with *sh-Control* MCF7 and T-47D cells (Additional file 6). We confirmed elevated expression of *APOBEC3B* in *sh-UNC5A* compared with *sh-Control* MCF7 cells (Fig. 4b). Based on the gene functional analysis using DAVID, genes differentially expressed in *UNC5A* knockdown MCF7 and T47-D cells were an integral part of the plasma membrane and extracellular region (Fig. 4c and Additional file 7). It is interesting to note that 167 DEGs in MCF7 cells were significantly overrepresented in sequence-specific transcription factor DNA binding activity (Fig. 4c). *UNC5A* knockdown also had a significant effect on E2-regulated gene expression, particularly in MCF7 cells. A total of 434 genes were recognized as undergoing significant changes in gene expression by E2 in *sh-UNC5A* MCF7 cells, whereas only 21 genes were identified as DEGs in *sh-UNC5A* T-47D cells (Fig. 4a and Additional file 6). In *sh-UNC5A* MCF7 cells but not in *sh-Control* cells, E2-targeted genes were associated with negative regulation of cell proliferation, extracellular region, and histone demethylase activity (Fig. 4c). For example, E2 induced the expression of histone demethylases KDM4B and KDM7A but reduced the levels of UTY and ARID5B in *sh-UNC5A* but not in *sh-Control* MCF7 cells [33] (Additional file 6). JARID2, which regulates the polycomb complex and histone methyltransferases [34], was E2 inducible in *sh-UNC5A* but not in *sh-Control* MCF7 cells (Additional file 6). Furthermore, we found that 109 out of these 434 genes (Fig. 4d) were not regulated by E2 in *sh-Control* MCF7 cells (FDR > 0.5 and |FC| < 1.15), whereas their expression was under E2 control in *sh-UNC5A* MCF7 cells. These 109 genes are associated with positive regulation of gene expression and affect transcriptional regulation by RNA polymerase II (Fig. 4c). By contrast, no specific GO functions could be assigned to uniquely E2-regulated genes in *sh-UNC5A* T-47D cells. We note that the effect of UNC5A on E2-regulated genes is gene-specific since *sh-Control* and *sh-UNC5A* MCF7 cells showed similar levels of E2-regulated expression of TFF1 and only a modest effect on E2-regulated expression of GREB1, two commonly used genes to measure E2-inducible genes (Fig. 4e). Collectively, these results indicate a cell type-dependent role of UNC5A in controlling basal and E2-regulated gene expression with potential downstream effects ranging from plasma membrane composition to transcriptional output from RNA polymerase II.

### *UNC5A* knockdown results in nonclassical luminal/basal hybrid gene expression pattern

**Fig. 5:**
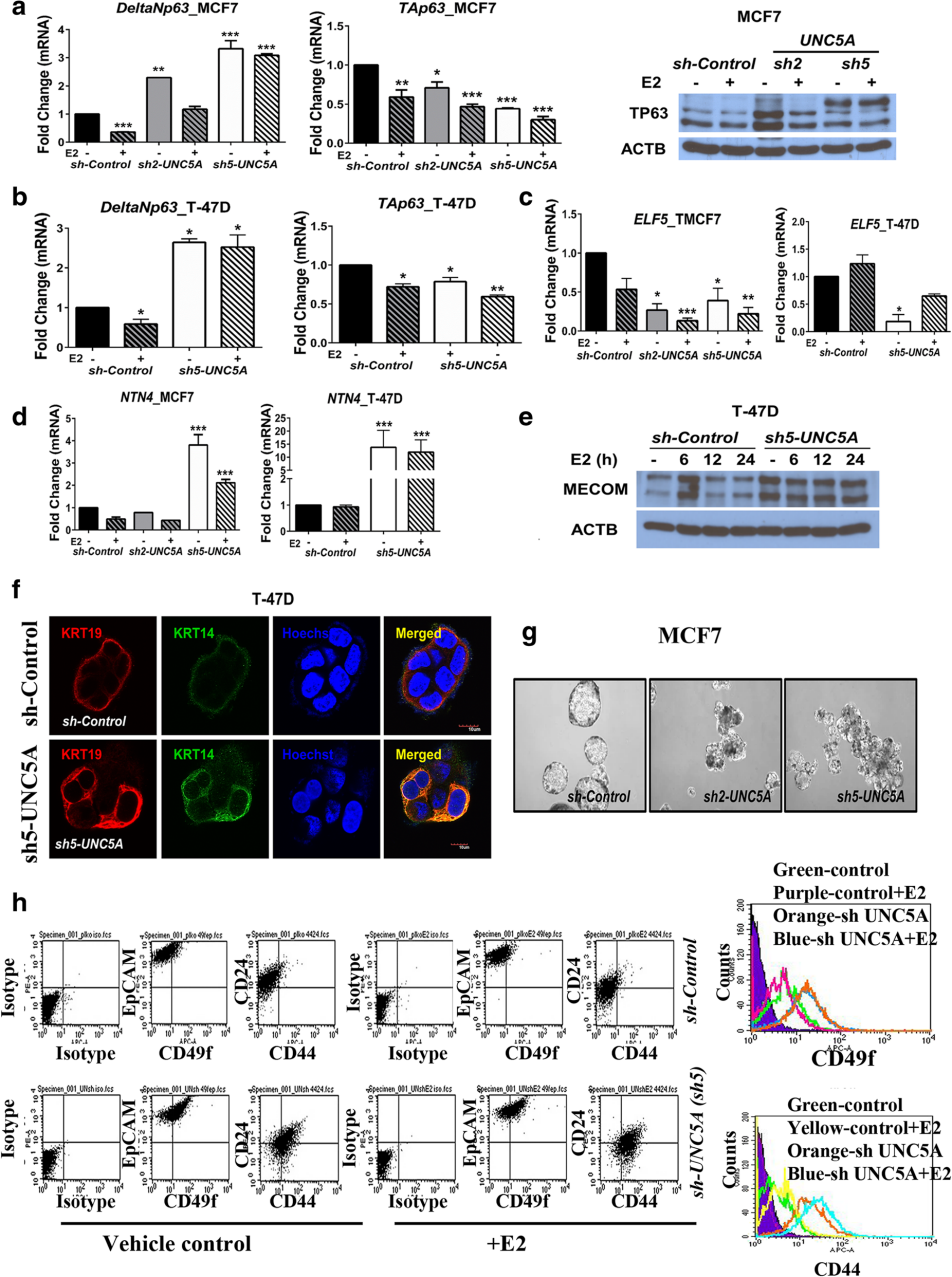
*UNC5A* knockdown results in luminal/basal hybrid and bipotent luminal progenitor phenotype. **a** *ΔNp63* levels are significantly elevated in *sh-UNC5A* MCF7 cells. Cells were treated with vehicle or estradiol (E2) for 3 h and qRT-PCR was used to measure *ΔNp63* and *TAp63* levels (mean ± SEM, *n* = 2). Data were analyzed as in Fig. 3e (*p < 0.05, **p < 0.01, and ***p < 0.001). TP63 protein levels in *sh-Control* and *sh-UNC5A* MCF7 cells treated with or without E2 for 24 h are shown on the right. TP63 is expressed as multiple isoforms and there appears to be isoform switching in *sh-UNC5A* cells compared with *sh-Control* cells. **b** *ΔNp63* and *TAp63* levels in *sh-Control* and *sh-UNC5A* T-47D cells. **c** *sh-UNC5A* cells have lower *ELF5* mRNA compared with *sh-Control* cells. Cells were treated with vehicle or E2 for 3 h and qRT-PCR was used to measure *ELF5*. **d** *UNC5A* knockdown leads to elevated *NTN4*. **e** UNC5A knockdown leads to elevated MECOM (EVI-1) expression in T-47D cells. Although *MECOM* levels were elevated in MCF7 cells upon *UNC5A* knockdown (Additional file 6), proteins were not detected by Western blotting. **f** *UNC5A* knockdown T-47D cells express both KRT14 (basal; green) and KRT19 (luminal; red) while *sh-Control* T-47D cells express luminal KRT19. Results for TMCF7 cells are shown in Additional file 8. **g** *sh-UNC5A* MCF7 cells form irregularly shaped mammospheres. **h** *UNC5A* knockdown MCF7 cells display phenotypic characteristics similar to luminal progenitor (CD49f^+^/EpCAM^+^)/cancer stem cells (CD44^+^/CD24^−^) compared with *sh-Control* cells. A histogram displaying CD49f and CD44 expression in different cell types is depicted on the right

*UNC5A* knockdown increased the levels of oncogenic *ΔNp63* isoform mRNA while simultaneously lowering the expression of tumor suppressive *TAp63* isoform [35] (Fig. 5a, b). *TP63* is an E2-repressed gene and ERα failed to repress ΔNp63 in *sh-UNC5A* clones with efficient *UNC5A* knockdown (*sh5-UNC5A* clones of MCF7 and T-47D; Fig. 5a, b). Overall, *ΔNp63* levels ± E2 treatment remained elevated in *sh-UNC5A* cells compared with *sh-Control* cells. RNA-seq studies showed lower expression of luminal/alveolar differentiation-associated *ELF5* but elevated expression of the pro-oncogenic *MECOM* (*EVI-1*) and lymphangiogenic *NTN4* [36–38] in *sh-UNC5A* cells compared with *sh-Control* cells (Additional file 6). Indeed, *ELF5* levels were significantly lower and *NTN4* levels were higher in *sh*-*UNC5A* cells compared with *sh-Control* cells (Fig. 5c, d). MECOM protein was undetectable in MCF7 cells but elevated in *sh-UNC5A* T-47D cells compared with *sh-Control* cells (Fig. 5e). In addition, while *sh-Control* cells expressed mainly KRT19, *sh-UNC5A* cells expressed either KRT14, KRT19, or both KRT14 and KRT19 (basal and luminal cytokeratins, respectively) [39] (Fig. 5f and Additional file 8). Bipotent luminal progenitor cells are KRT14 and KRT19 double-positive [40]. Note that *UNC5A* knockdown did not result in the morphologic features of epithelial to mesenchymal transition (EMT), nor did it result in the expression of EMT-associated genes such as *SNAI1*, *SNAI2*, *ZEB1*, or *ZEB2* (Additional file 6 and data not shown). However, we observed elevated expression of *ITGB6* (Integrin β6) in *sh*-*UNC5A* cells compared with *sh-Control* cells (Additional file 6); ITGB6 is pro-oncogenic and is induced during EMT of colon cancer cells [41].

Since ΔNp63 maintains stem cell phenotype and cancer cells with hybrid luminal/basal/mesenchymal characteristics display enhanced cancer stem cell (CSC) properties [35, 42], we used mammosphere assays and flow cytometry to characterize *sh-Control* and *sh-UNC5A* MCF7 cells for stemness. While mammospheres of *sh-Control* were well organized, *sh-UNC5A* cells formed irregular mammospheres (Fig. 5g). In addition, while *sh-Control* cells were predominantly CD49f (ITGA6)^−^/EPCAM^+^, a subpopulation of *sh-UNC5A* cells showed CD49f^+^/EPCAM^+^ phenotype (Fig. 5h). CD49f^+^/EPCAM^−^, CD49f^+^/EPCAM^+^, and CD49f^−^/EPCAM^+^ cells display stem/basal, luminal progenitor, and differentiated/mature features, respectively [43]. *Sh-Control* cells showed CD44^−^/CD24^+^ non-CSC phenotype whereas *sh-UNC5A* cells acquired the features of CSCs as evident from the presence of CD44^+^/CD24^+^ and CD44^+^/CD24^−^ cells [44]. Furthermore, *sh-UNC5A* MCF7 cells expressed significantly higher levels of stemness-associated *SOX2* [45] (Additional file 6).

### *UNC5A* knockdown results in elevated EGFR expression and AKT activity

**Fig. 6:**
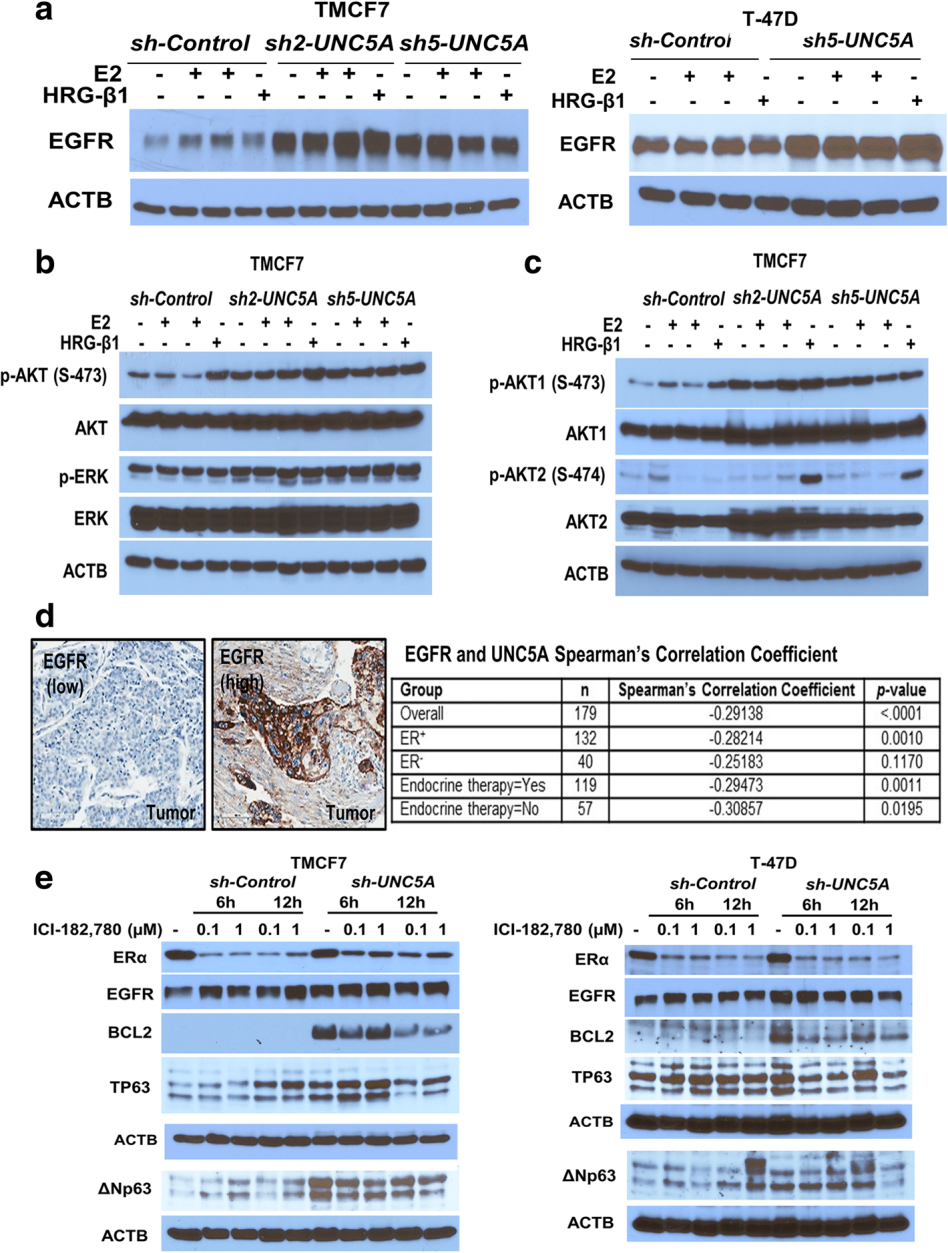
*UNC5A* knockdown results in elevated epidermal growth factor receptor (EGFR) and phospho-AKT. **a** EGFR protein levels in *sh-Control* and *sh-UNC5A* TMCF7 and T-47D cells. **b** Phospho-AKT and phospho-ERK levels in *sh-Control* and *sh-UNC5A* TMCF7 cells with or without estradiol (E2) or HRG-β1 (20 ng/ml) treatment. Cells were treated with vehicle, E2 for 5 and 15 min, and HRG-β1 for 15 min. **c** Phospho-AKT1 and phospho-AKT2 levels in *sh-Control* and *sh-UNC5A* TMCF7 cells. **d** Representative EGFR staining pattern in breast tumors with low or high expression (left, scale bar = 100 μm). Summary of the correlation analysis between EGFR and UNC5A from breast cancer TMA using Spearman’s correlation coefficient (right). UNC5A and EGFR expression was inversely correlated (*p* < 0.001, *n* = 179). **e** Protein levels of estrogen receptor (ER)α, EGFR, BCL2, and TP63 in *sh-Control* and *sh-UNC5A* TMCF7 (left) and T-47D (right) cells after treatment with ICI-182,780 (Fulvestrant)

Two of our observations and one prior report prompted us to investigate whether *UNC5A* knockdown is associated with altered activity of EGFR, which could explain the effects of UNC5A on E2-regulated gene expression. First, we observed enhanced basal ERE-luciferase activity in *sh-UNC5A* cells, suggesting ligand-independent activity of ERα which typically involves growth factor receptor–ERα crosstalk [46]. EGFR is forefront in this crosstalk as it can alter ERα cistrome and ERα-regulated gene expression [47]. Second, *sh-UNC5A* cells showed luminal/basal hybrid phenotype, and EGFR activation is common in cells with basal phenotype [48]. Third, a recent study showed that NTN1, in the absence of UNC5A, increases EGFR at the post-translational level [49]. We first measured EGFR levels in *sh-Control* and *UNC5A* knockdown cells. EGFR protein but not mRNA levels were significantly higher in *sh-UNC5A* cells compared with *sh-Control* cells (Fig. 6a and data not shown). AKT and ERK are the two major kinases activated downstream of EGFR that can increase ligand-independent activity of ERα [1]. We determined whether *UNC5A* knockdown had an effect on vehicle, E2-regulated, and HRGβ1-induced activation of these kinases. *sh-UNC5A* cells showed robust activation of AKT as measured by pAKT-S473 levels but not ERK (Fig. 6b). We recently reported that AKT1 but not AKT2 is active in MCF7 cells [17]. Immunoblotting using isoform-specific phospho-antibodies showed upregulation of pAKT1 but not pAKT2 in *sh-UNC5A* cells compared with *sh-Control* cells (Fig. 6c). These results indicate a negative relationship between EGFR and UNC5A expression in cell line models. Similarly, UNC5A and EGFR expression showed negative correlation in breast tumor samples when the analyses included all samples or only ER^+^ samples (Fig. 6d).

We next investigated the role of ERα in negative crosstalk between UNC5A and EGFR/TP63. Treatment of cells with ICI-182,780 results in degradation of ERα and, consequently, elevated expression of genes typically repressed by ERα. Indeed, treatment of *sh-Control* MCF7 and T-47D cells caused degradation of ERα with a concomitant increase in TP63 (Fig. 6e). Interestingly, ICI-182,780 treatment did not have an effect on EGFR but reduced the level of TP63 in *sh-UNC5A* cells (Fig. 6e). Similar to TP63, elevated expression of BCL2 upon *UNC5A* knockdown is ERα-dependent as its levels were lower in ICI-182,780-treated cells compared with untreated *sh-UNC5A* cells (Fig. 6e). Thus, while elevated EGFR levels in *sh-UNC5A* cells are ERα-independent, ΔNp63 and BCL2 upregulation in these cells is at least partially ERα-dependent.

### *sh-UNC5A* cells form metastatic tumors independent of E2 supplementation

MCF7 cells form nonmetastatic tumors in female nude mice when injected with matrigel or when supplemented with E2 pellets, although there is less uniformity between the sizes of tumors between animals [16]. We had previously reported in the MDA-MB-231 model that cell lines derived from tumors that develop in the mammary fat pad upon implantation of parental cells show enhanced and uniform tumorigenicity upon re-implantation [50]. We used this approach to increase uniformity in tumorigenicity and generated TMCF7 *sh-Control*, *sh2-UNC5A*, and *sh5-UNC5A* cells. As with MCF7 cells, *sh-UNC5A* TMCF7 cells showed elevated BCL2 and *ΔNp63* compared with *sh-Control* cells (Additional file 8). *sh-UNC5A* but not *sh-Control* TMCF7 cells displayed KRT14/KRT19 double-positive phenotype (Additional file 8). A large subpopulation of *sh-UNC5A* TMCF7 cells was of the CD44^+^/CD24^+^ and CD49f^+^/EPCAM^+^ phenotype compared with *sh-Control* cells (Additional file 8), and mammospheres formed by these cells were irregular compared to mammospheres from *sh-Control* cells (Additional file 8). In addition, *sh-UNC5A* TMCF7 cells expressed significantly higher levels of *SOX2* despite maintaining ERα expression (Additional file 8). Consistent with RNA-seq data (Additional file 6), a large subpopulation of *sh-UNC5A* TMCF7 cells were ITGB6^+^ compared with *sh-Control* cells (Additional file 8).

**Fig. 7:**
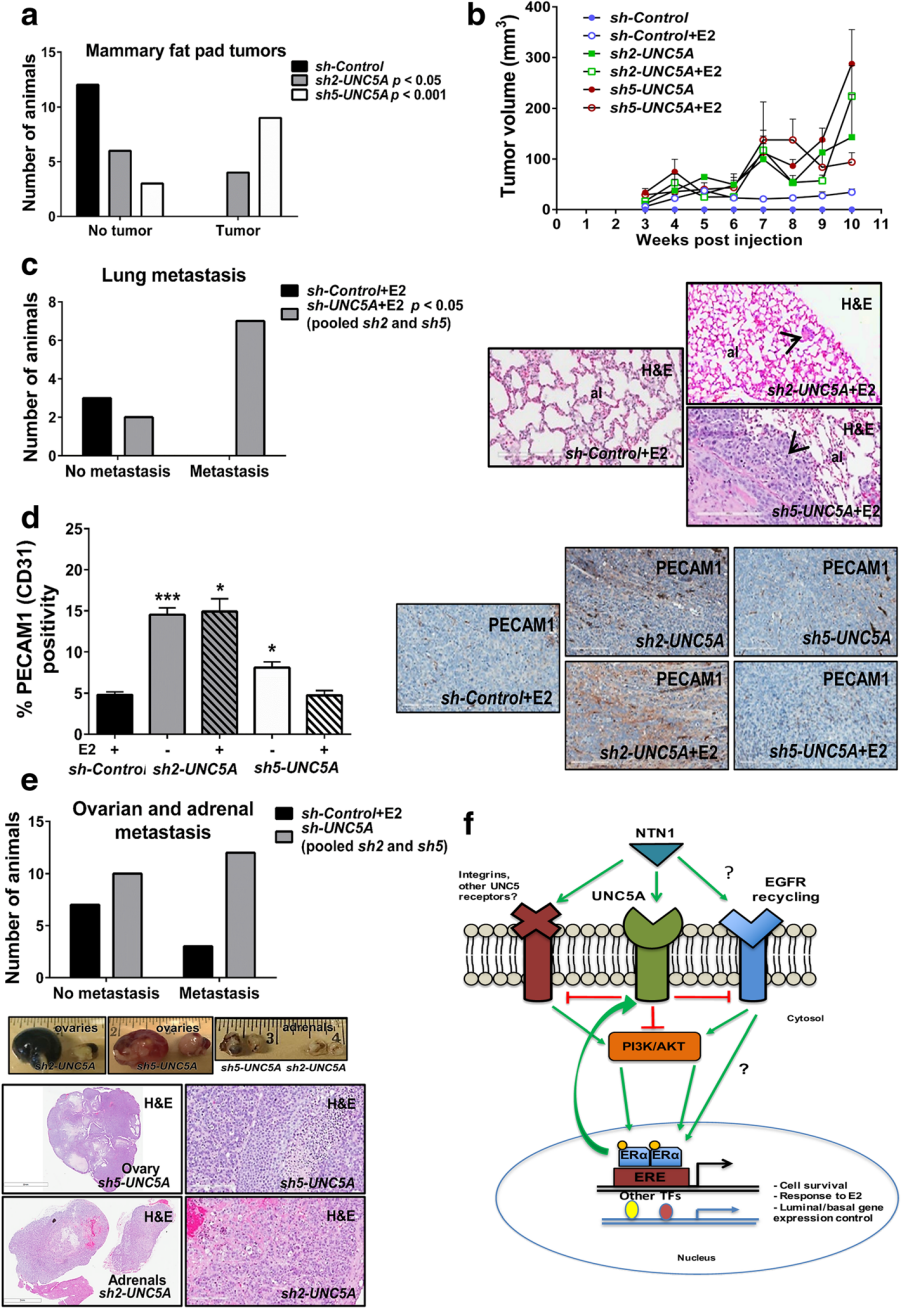
*UNC5A* knockdown cells form tumors independent of E2 supplementation. **a** The number of animals with tumors after injection of *sh-Control*, *sh2-* and *sh5-UNC5A* TMCF7 cells in the absence of implanted estradiol (E2) pellet. Number of *sh2-UNC5A* (*p* < 0.05) and *sh5-UNC5A* (*p* < 0.001) mammary fat pad tumor-positive mice at the time of euthanasia (11 weeks postinjection). **b** Tumor volume in animals injected with *sh-Control*, *sh2-UNC5A*, or *sh5-UNC5A* TMCF7 cells with or without E2 pellets (mean ± SEM). **c** Lung metastasis pattern in animals injected with *sh-Control* or *sh-UNC5A* cells into the mammary fat pad in the presence of implanted E2 pellets. The number of animals with and without lung metastasis is shown on the left (*p* < 0.05), whereas representative lung sections stained with hematoxylin and eosin (H&E) from *sh-Control*, *sh2-*, and *sh5-UNC5A* TMCF7 cell inoculated mice with an E2 pellet is shown on the right. Arrowheads point to metastatic tumor cells (al, alveolus; scale bar = 200 μm). Data were analyzed using the Fisher’s exact test (two-tailed). Lungs from only three *sh-Control* cell injected animals were examined because we had previously shown a lack of lung metastasis in animals injected with TMCF7 cells [16]. **d** *sh-UNC5A* TMCF7 cell-derived tumors show significant levels of angiogenesis. PECAM1 staining was performed to measure endothelial cells in tumors. The number of PECAM1^+^ cells in at least 12 fields per tumor is shown on the left, whereas representative PECAM1 staining pattern is shown on the right (scale bar = 200 μm; *p < 0.05 and ***p < 0.001). **e** *UNC5A* knockdown cells show enhanced metastases to ovaries and adrenal glands. The number of animals with metastases to ovaries and adrenal glands is shown on the left, whereas the gross appearance of ovaries and adrenal glands of *sh-UNC5A* TMCF7 cells injected via the intracardiac route and the H&E staining pattern of ovary and adrenal gland with metastasis are shown on the right. Scale bars = 3 mm and 200 μm. **f** Model depicting crosstalk between UNC5A, epidermal growth factor receptor (EGFR), and E2 signaling. Unliganded UNC5A likely inhibits E2 signaling, which may be reversed upon binding of NTN1. Question marks indicates unknown mechanisms of regulation. ER, estrogen receptor

A significant number of mice injected with *sh-UNC5A* cells but not *sh-Control* cells developed tumors in the absence of E2 pellets (Fig. 7a). The size of these tumors was larger than tumors in animals injected with *sh-Control* cells in the presence of E2 pellet (Fig. 7b). While none of the animals injected with *sh-Control* cells developed lung metastasis, consistent with our previous study with TMCF7 cells [16], animals that received *sh-UNC5A* cells showed lung metastasis (Fig. 7c). We next examined whether *sh-Control* cell- and *sh-UNC5A* cell-derived tumors differ in angiogenesis because of the differences in *NTN4* expression between *sh-Control* and *sh-UNC5A* cells noted in Fig. 5. *sh-UNC5A* cell-derived tumors contained higher numbers of PECAM1^+^ cells compared with *sh-Control* cell-derived tumors (Fig. 7d), suggesting enhanced angiogenesis in the absence of *UNC5A*.

To determine whether *UNC5A* knockdown enhanced the multiorgan homing capacity of tumor cells, *sh-Control* and *sh-UNC5A* cells were injected via the intracardiac route into animals supplemented with E2 pellets. Autopsies within 17 weeks of injection revealed growth of tumor cells in ovaries and adrenal glands at a higher frequency in animals injected with *sh-UNC5A* cells compared with *sh-Control* cells (Fig. 7e). Histological analysis revealed a severe disruption of the normal architecture of both organs (Fig. 7f). Ovaries were devoid of follicles and corpora lutea, and the very few remaining were undergoing atresia and degeneration. Likewise, adrenals lost a clear differentiation between the cortex and medulla zones with hemorrhagic areas and high vacuolization even in areas where the capsule is still preserved. Spleens of animals that received *sh-UNC5A* cells showed extramedullary hematopoiesis with enhanced myeloid and erythroid elements and megakaryocytes causing distention of the spleen in the red pulp area (data not shown). Overall, the results presented above clearly indicate the role of UNC5A in regulating the metastasis of ER^+^ tumors and its loss of expression leading to E2-independent growth both in vitro and in vivo.

## Discussion

UNC5A is a transmembrane receptor that generates cell survival or death signals in a ligand-dependent manner [10]. UNC5A and NTN1 are described as tumor suppressor and oncogene, respectively, in breast cancer [51, 52]. However, signaling pathways that control their expression to alter the balance between UNC5A and NTN1 are unknown. Analyses of TCGA dataset showed elevated expression of UNC5A in luminal breast cancers, and NTN1 overexpression in TNBCs and E2 could further enhance luminal expression of UNC5A (Fig. 1). Thus, the UNC5A–NTN1 signaling axis is likely tilted more towards UNC5A-activated signals in ERα^+/^PR^+^ breast cancers and NTN1-generated signals in TNBC/ER^−^ tumors. Consistent with this possibility, UNC5A expression was prognostic in ER^+^/PR^+^/HER2^−^ breast cancers but not in ER^−^ tumors, suggesting its critical role in ER^+^/PR^+^/HER2^−^ breast cancers. A subgroup of women with ER^+^/PR^+^ breast cancers develop recurrence, and molecular assays such as the Recurrence Score and Breast Cancer Index are helping to identify ER^+^/PR^+^ breast cancer patients requiring hormonal and/or chemotherapy [53]. UNC5A, possibly in combination with EGFR, could be developed as a biomarker to identify such patients [4].

Molecular events causing variable UNC5A expression in ER^+^ tumors are unknown. *UNC5A* is a TP53-inducible gene, and TP53 is infrequently mutated in ER^+^/PR^+^ breast cancer [2, 54]. Deregulated p53 activity instead of mutations may lead to loss of UNC5A expression in ER^+^/PR^+^ tumors, although this remains to be investigated. In addition, there is potential for p53 to control UNC5A activity since we noted a differential effect of *UNC5A* knockdown on proliferation in wild-type p53 containing MCF7 cells compared with mutant p53 containing T-47D cells, although knockdown had a similar effect on BCL2 and TP63 expression in both cell lines. cBioPortal analyses revealed frequent missense and truncating mutations in *UNC5A* [55]. Additionally, *UNC5A* expression is regulated through allele-specific DNA methylation [56]. Thus, mutations and DNA methylation could be other mechanisms leading to inactivation/silencing of *UNC5A* during breast cancer progression.

One of the consequences of reduced UNC5A expression is significant changes in basal gene expression and altered ERα signaling. GO analyses revealed a specific effect of *UNC5A* knockdown on a network of transcription factors including the stem cell-associated transcription factor *SOX2*, which may be a reason for the altered expression of 20% of genes in *sh-UNC5A* MCF7 cells and 7% in *sh-UNC5A* T-47D cells compared with *sh-Control* cells (Fig. 4). It is interesting that, in both cell lines, *UNC5A* knockdown affected the expression of genes linked to the plasma membrane and extracellular region composition (Fig. 4), which can explain the aggressive growth characteristics of *sh-UNC5A* compared with *sh-Control* MCF7 cells in vivo. We also observed a distinct effect of UNC5A on E2-regulated gene expression, with several genes gaining E2-regulated gene expression (Fig. 4). These results suggest a role for UNC5A in restricting the activity of unliganded ERα in a gene-specific manner, which could involve the following mechanisms. One possibility is the direct effect of UNC5A-activated signals on chromatin organization since we observed an effect of *UNC5A* knockdown on the histone demethylation network in E2-treated cells (Fig. 4). *UNC5A* knockdown increased the E2-inducible expression of KDM4B, which is a master regulator of ERα activity [57]. Elevated KDM4B in *sh-UNC5A* cells could further amplify the E2 signaling axis as evident from more than 400 genes gaining E2-regulated expression in *sh-UNC5A* cells. Robustness at which *UNC5A* knockdown altered BCL2 expression further suggests a direct link between UNC5A and chromatin organization. This drastic effect of *UNC5A* knockdown on BCL2 expression is reminiscent of permanent gene expression changes observed upon transient knockdown of DNMT1 [38]. However, we did not observe an effect of *UNC5A* knockdown on the expression of any *DNMTs*, although there was a modest but statistically significant effect on *TET1* and *TET3* which antagonize DNMTs (Additional file 6). *UNC5A* knockdown may have an effect on histone acetylation/deacetylation since *sh-UNC5A* MCF7 cells expressed significantly higher levels of the epigenetic regulator *HDAC9* compared with *sh-Control* cells (Additional files 6). The second possibility is the involvement of *ELF5*. ELF5 suppresses E2 sensitivity by reducing the expression of *ESR1* and the pioneer factors *FOXA1* and *GATA3* [36]. ERα, FOXA1, and GATA3 constitute a positive lineage-restricted hormone responsive regulatory loop in luminal cells [58]. We observed the effect of *UNC5A* knockdown on *ELF5* expression, and reduced *ELF5* expression in *sh-UNC5A* MCF7 cells correlated with elevated *GATA3* expression (Additional file 6). The third possibility is the involvement of AKT. *UNC5A* knockdown caused upregulation of activated AKT, which confers ligand-independent activity to ERα [59]. The fourth possibility involves ERα–EGFR crosstalk since *UNC5A* knockdown cells contained higher levels of EGFR protein (Fig. 7), and EGF through EGFR has been shown to alter ERα cistrome and transcriptome [60].

*UNC5A* knockdown in MCF7 cells resulted in a hybrid phenotype with cells expressing luminal (ER, *PGR*), myoepithelial (TP63), and stem cell markers (*SOX2*), which in part is due to altered ERα signaling. Recent studies have identified similar hybrid cells in primary breast cancers, potentially generated through Notch-Jagged signaling [42, 61]. Based on cell surface marker profiles and KRT14/KRT19 expression patterns in *sh-UNC5A* cells, we propose that the gradual loss of *UNC5A* results in cancer cells acquiring hybrid phenotype without expressing classic markers of EMT. Since there is still a controversy related to in-vivo detection of cancer cells with EMT features, it is possible that primary tumors contain cells with hybrid phenotype which functionally behave like cancer cells with EMT features. Characterizing primary tumors for ER, PR, UNC5A, EGFR, and additional basal cell markers would allow the detection of such hybrid cells. Collectively, results presented in this study provide novel insights into pathways that restrict ERα signaling and metastatic progression of ERα^+^ breast cancer, which potentially involves luminal to luminal/basal hybrid conversion due to an aberrant DR pathway.

## Conclusions

In this study, we demonstrate an unexpected role for the dependence receptor UNC5A in regulating ERα activity and restricting the expression of basal cell-enriched genes in luminal cells. Progressive loss of *UNC5A* expression could result in ERα-positive luminal cells acquiring basal features including the expression of ΔNp63, *SOX2*, and EGFR, while maintaining ERα expression. These results describe the role of UNC5A in controlling plasticity of luminal breast cancer. Therefore, UNC5A, ERα, and EGFR could be developed as markers to identify luminal breast cancers with a potential for subtype conversion.

## Additional files

Additional file 1: Tables that describe antibodies and primers used in the study. (DOCX 121 kb)

Additional file 2: E2-regulated expression of UNC5A in various cell types extracted from published studies using NURSA data resource. (XLSX 13 kb)

Additional file 3: Description of patients and characteristics of their tumors (*n* = 221). (DOCX 98 kb)

Additional file 4: Summary of results of overall survival: univariate and multivariate analyses on the UNC5A *H* score category. (DOCX 96 kb)

Additional file 5: The effect of ICI-182,780 (Fulvestrant) treatment on *UNC5A* knockdown cells and Western blot showing UNC5A knockdown by CRISPR in MCF-7 cells. (PSD 3346 kb)

Additional file 6: Summary of results of RNA*-*seq of *sh-Control* and *sh5-UNC5A* clones of MCF7 and T-47D cells. Various comparisons are shown. (XLSX 859 kb)

Additional file 7: Pathways analyses using DAVID of differentially expressed genes under different conditions and in different cell types. (XLSX 139 kb)

Additional file 8: Characterization of TMCF7 cells with and without UNC5A knockdown for stemness and luminal/basal hybrid features. (PSD 50580 kb)

## Abbreviations

ACTB: Beta-actin
AKT: v-AKT murine thymoma viral oncogene
APOBEC3B: Apolipoprotein B mRNA-editing enzyme
BCL2: B cell leukemia/lymphoma 2
CCS: Charcoal-dextran treated fetal calf serum
ChIP: Chromatin immunoprecipitation
CSC: Cancer stem cell
DEG: Differentially expressed gene
DNMT1: DNA methyltransferase 1
DR: Dependence receptor
E2: Estradiol
EGFR: Epidermal growth factor receptor
ELISA: Enzyme-linked immunosorbent assay
EMT: Epithelial to mesenchymal transition
ER: Estrogen receptor
ERE: Estrogen response element
ERK: Extracellular signal-regulated kinase
FC: Fold change
FDR: False discovery rate
GEO: Gene expression omnibus
GO: Gene ontology
H&E: Hematoxylin and eosin
HRG: Heregulin
MEM: Minimum essential media
NTN: Netrin
OHT: 4-Hydroxy tamoxifen
PBS: Phosphate-buffered saline
PR: Progesterone receptor
qRT-PCR: Quantitative reverse transcription polymerase chain reaction
shRNA: Short hairpin RNA
TCGA: The Cancer Genome Atlas
TMA: Tissue microarray
TNBC: Triple negative breast cancer
UNC5A: Unc-5 netrin receptor A
