# Supplementary material for: Dependence receptor UNC5A restricts luminal to basal breast cancer plasticity and metastasis
Source: Breast Cancer Res. 2018 May 2;20:35. doi: 10.1186/s13058-018-0963-5 (PMC5932758; doi:10.1186/s13058-018-0963-5)
Supplement: Supplementary file 1 — Tables that describe antibodies and primers used in the study. (DOCX 121 kb) [file 13058_2018_963_MOESM1_ESM.docx]

**Antibodies Table**

| **Peptide/protein target** | **Name of Antibody** | **Manufacturer, catalog #, and/or name of individual providing the antibody** |
| --- | --- | --- |
| UNC5A | Rb pAB to Unc5a | Abcam - ab81165 |
| TP63 | p63 (4A4) | Santa Cruz - sc-8431 |
| β-ACTIN | Monoclonal Anti-β-Actin Clone AC15 | Sigma Life Sciences - Cat # A5441 |
| BCL2 | Purified mouse Anti-Bcl-2 | BD transduction Laboratories - Cat # 610538 |
| EGFR | EGFR (1005) | Santa Cruz Biotechnology - sc-03 |
| Estrogen Receptor alpha | ERα (HC-20) | Santa Cruz Biotechnology - sc-543 |
| Estrogen Receptor alpha | ERα (F-10) | Santa Cruz Biotechnology - sc-8002 |
| Phospho-Estrogen Receptor alpha S118 | pERαS118 | Cell Signaling 2511 |
| Phospho-Estrogen Receptor alpha S167 | pERαS167 | QED Biosciences, INC 43073 |
| Total AKT | Akt rabbit Ab | Cell Signaling Technologies - Cat #9272BC |
| p-AKT (S473) | p-Akt (S473) Rabbit Ab | Cell Signaling Technologies - Cat #9271S |
| p-ERK | p44/42 MAP Kinase Antibody | Cell Signaling Technologies - Cat #9102 |
| Total ERK | ERK 1 (K-23) | Santa Cruz Biotechnology - sc-94 |
| AKT1 | Akt1 (2H10) mouse mAb | Cell Signaling Technologies - Cat #2967S |
| AKT2 | Akt2 (L7B2) mouse mAb | Cell Signaling Technologies - Cat #5239S |
| p-AKT1 | P-Akt1 (S473) (DF10) Rabbit mAb (Akt1 specific) | Cell Signaling Technologies - Cat #9018BC |
| p-AKT2 | P-AKt2 (S474) (D3H2) Rabbit mAB (Akt2 specific) | Cell Signaling Technologies - Cat #8599S |
| MECOM (EVI-1) | Evi-1 (C50E12) | Cell Signaling Technologies - Cat #2593S |
|  |  |  |
| Cytokeratin 14 | Anti-Cytokeratin 14 [LL002] | Abcam - ab7800 |
| Cytokeratin 19 | Anti-Cytokeratin 19 antibody [EP1580Y] | Abcam - ab52625 |
|  |  |  |
| CD24 | PE Mouse Anti-Human CD24 | BD Pharmigen - Cat # 555428 |
| CD44 | APC Mouse Anti-Human CD44 | BD Pharmigen - Cat # 559942 |
| IgG-Isotype Control | PE Mouse IgG1 κ Isotype Control | BD Pharmigen - Cat # 555749 |
| IgG-Isotype Control | APC Mouse IgG2a κ Isotype Control | BD Pharmigen - Cat # 555576 |
| EPCAM | CD 326 (EpCAM)-PE | MACS Miltenyl Biotec - Cat # 130-091-253 |
| Integrin β6 | anti-hIntegrin β6 APC conjugated mouse IgG2B | R & D systems - FAB4155A |
| Integrin α6 (CD49f) | anti-h/M/BIntegrin α6 APC conjugated rat IgG2A | R & D systems - FAB13501A |
| **Δ**Np63 | Purified anti-p63 (**Δ)** antibody | Biolegend- Cat #619001 |

| **Primers used in this study** | |
| --- | --- |
|  | Primer Sequence (5’- 3’) |
| *ACTB* | Forward: AAT-GRG-GCC-GAG-GAC-TTT-GAT-TGC  Reverse: AGG-ATG-GCA-AGG-GAC-TTC-CTG-TAA |
| *SOX2* | Forward: CAC-ATG-AAG-GAG-CAC-CCG-GAT-TAT  Reverse: GTT-CAT-GTG-GGC-GAA-CTG-TCC-AT |
| *TAp63* | Forward: GTG-CGA-CAA-ACA-AGA-TTG-AGA-TTA-G  Reverse: TGT-TCA-GGA-GCC-CCA-GGT-T |
| *∆Np63* | Forward: AAA-GGA-CAG-CAG-CAT-TGA-TCA-A  Reverse: TGT-TCA-GGA-GCC-CCA-GGT-T |

**Quantitative RT-PCR primers (qPCR)**

**TaqMan probes**

| *ACTB* | Hs01060665_g1 |
| --- | --- |
| *BCL2* | Hs00608023_m1 |
| *APOBEC3B* | Hs00358981_m1 |
| *ELF5* | Hs01063022_m1 |
| *NTN4* | Hs00221915_m1 |
| *PGR* | Hs01556702_m1 |
| *UNC5A* | Hs00293338_m1 |

**ChIP primers**

|  | Primer Sequence (5’- 3’) |
| --- | --- |
| *BCL2* | Forward: CAC-CTG-TGG-TCC-ACC-TGA-C  Reverse: CTG-AAGAGC-TCC-TCC-ACC-AC |
| *GREB1* | Forward: TGC-TTC-TGA-AGG-GCA-GAG-CTG-ATA  Reverse: TAG-GTC-CAG-AAT-GAC-CCA-GTT-GCC-A |
| *UNC5A* | Forward: CCC-GCA-GCT-TCA-GTC-CAG-CCC  Reverse: CCT-GCA-AAG-AGA-GCC-CAA-ACT |
