# Supplementary material for: Dependence receptor UNC5A restricts luminal to basal breast cancer plasticity and metastasis
Source: Breast Cancer Res. 2018 May 2;20:35. doi: 10.1186/s13058-018-0963-5 (PMC5932758; doi:10.1186/s13058-018-0963-5)
Supplement: Supplementary file 3 — Description of patients and characteristics of their tumors (n = 221). (DOCX 98 kb) [file 13058_2018_963_MOESM3_ESM.docx]

**Description of the patients and characteristics of their tumors (n=221)**

| **Characteristic** | **Have UNC5**  **(N=196)** | **Missing UNC5**  **(N=25)** | **p-value*** |
| --- | --- | --- | --- |
| Age at Diagnosis, *y* |  |  | 0. 0739 |
| Mean (SD) | 58.0 (15.2) | 63.8 (13.4) |  |
| Median | 57.0 | 65.0 |  |
| Range | 27.00-88.00 | 36.00-83.00 |  |
| Race, *n(%)* |  |  | 0. 0050 |
| White | 146 (74.49%) | 25 (100.0%) |  |
| African American | 48 (24.49%) | 0 |  |
| Asian | 1 ( 0.51%) | 0 |  |
| Other | 1 ( 0.51%) | 0 |  |
| PR Status, *n(%)* |  |  | 0. 4762 |
| Negative | 67 (34.18%) | 7 (28.00%) |  |
| Positive | 109 (55.61%) | 16 (64.00%) |  |
| Unknown | 20 (10.2%) | 2 ( 8.00%) |  |
| ER Status, *n(%)* |  |  | 0.7784 |
| Negative | 44 (22.45%) | 5 (20.00%) |  |
| Positive | 144 (73.47%) | 19 (76.00%) |  |
| Not Done | 8 ( 4.08%) | 1 ( 4.00%) |  |
| HER-2/neu, *n(%)* |  |  | 0. 7359 |
| Negative | 110 (56.12%) | 12 (48.00%) |  |
| Positive | 32 (16.33%) | 2 ( 8.00%) |  |
| Unknown/Not Done | 54 (27.6%) | 11 (44.0%) |  |
| Tumor Grade, *n(%)* |  |  | 0. 9580 |
| I | 45 (22.96%) | 5 (20.00%) |  |
| II | 84 (42.86%) | 11 (44.00%) |  |
| III | 47 (23.98%) | 6 (24.00%) |  |
| Unknown | 20 (10.20%) | 3 (12.00%) |  |
| T Stage, *n(%)* |  |  | 0. 0184 |
| T0 | 0 | 0 |  |
| T1 | 97 (49.49%) | 19 (76.00%) |  |
| T2 | 76 (38.78%) | 3 (12.00%) |  |
| T3 | 14 ( 7.14%) | 1 ( 4.00%) |  |
| T4 | 8 ( 4.08%) | 2 ( 8.00%) |  |
| TX/Unknown | 1 ( 0.51%) | 0 |  |
| N Stage, *n(%)* |  |  | 0. 5475 |
| N0 | 108 (55.10%) | 18 (72.00%) |  |
| N1 | 58 (29.59%) | 5 (20.00%) |  |
| N2 | 18 ( 9.18%) | 1 ( 4.00%) |  |
| N3 | 4 ( 2.04%) | 0 |  |
| NX/Unknown | 8 ( 4.08%) | 1 ( 4.00%) |  |
| M Stage, *n(%)* |  |  | 1.0000 |
| M0 | 169 (86.22%) | 23 (92.00%) |  |
| M1 | 7 ( 3.57%) | 1 ( 4.00%) |  |
| MX/Unknown | 20 (10.20%) | 1 ( 4.00%) |  |
| Endocrine Therapy, *n(%)* |  |  | 0. 9900 |
| Yes | 131 (66.84%) | 17 (68.00%) |  |
| No | 62 (31.63%) | 8 (32.00%) |  |
| Unknown | 3 (1.5%) | 0 |  |
| Death, *n(%)* |  |  | 0. 6083 |
| Yes | 73 (37.24%) | 8 (32.00%) |  |
| No | 123 (62.76%) | 17 (68.00%) |  |
| Recurrence, *n(%)* |  |  | 0. 9059 |
| Yes | 76 (38.78%) | 10 (40.00%) |  |
| No | 120 (61.22%) | 15 (60.00%) |  |
| Follow-up, *y*, median (95% CI) | 10.3 (0.04, 21.8) | 10.2 (1.2, 12.9) | 0.6380 |
| Overall survival, *y*, median (95% CI) | 13.1 (11.7, NE) | 11.3 (11.2, NE) | 0.7578 |
| Disease-free survival, *y*, median (95% CI)** | 11.1 (9.6, 12.4) | 11.4 (4.0, 11.4) | 0.9949 |
