# Supplementary material for: Dependence receptor UNC5A restricts luminal to basal breast cancer plasticity and metastasis
Source: Breast Cancer Res. 2018 May 2;20:35. doi: 10.1186/s13058-018-0963-5 (PMC5932758; doi:10.1186/s13058-018-0963-5)
Supplement: Supplementary file 4 — Summary of results of overall survival: univariate and multivariate analyses on the UNC5A H score category. (DOCX 96 kb) [file 13058_2018_963_MOESM4_ESM.docx]

**Summary of results for Overall Survival.**

**Univariate analyses on the UNC5 H-score category**

| **Obs** | **analtype** | **rankscore** | **Num of Events** | **Num Censored** | **Median (95% CI)** | **Logrank p-value** |
| --- | --- | --- | --- | --- | --- | --- |
| **1** | OS | _ | 73 (37.24) | 123 (62.76) |  | 0.0421 |
| **2** | OS | Low | 38 (44.71) | 47 (55.29) | 11.74 ( 8.53, 12.40) |  |
| **3** | OS | High | 35 (31.53) | 76 (68.47) | 15.37 (13.09, . ) |  |

**Multivariable analysis for Overall Survival (N=188)**

| **Variable** | **Comparison*** | **HR (95% CI)** | **p-value**** |
| --- | --- | --- | --- |
| Tumor Grade | Grade 1 vs Grade 2 | 0.63 (0.29, 1.34) | 0.1430 |
|  | Grade 1 vs Grade 3 | 0.40 (0.18, 0.90) |  |
|  | Grade 1 vs Unknown | 0.65 (0.23, 1.81) |  |
|  | Grade 2 vs Grade 3 | 0.63 (0.35, 1.14) |  |
|  | Grade 2 vs Unknown | 1.04 (0.45, 2.40) |  |
|  | Grade 3 vs Unknown | 1.64 (0.68, 3.92) |  |
| T Stage | T0/1 vs T2 | 0.75 (0.41, 1.35) | 0.3283 |
|  | T0/1 vs T3/4 | 0.55 (0.24, 1.28) |  |
|  | T0/1 vs TX | 0.20 (0.02, 1.76) |  |
|  | T2 vs T3/4 | 0.74 (0.35, 1.55) |  |
|  | T2 vs TX | 0.26 (0.03, 2.26) |  |
|  | T3/4 vs TX | 0.34 (0.04, 3.20) |  |
| N Stage | N+ vs N0 | 1.42 (0.80, 2.52) | 0.2361 |
| UNC5 H-score Category | Low vs High | 1.70 (1.02, 2.83) | 0.0403 |

*referent group listed second, **from Wald Chi-square test
